# An engineered adipose formulation decreases hepatic inflammation and fibrosis in a rodent model of metabolic dysfunction-associated steatotic liver disease

**DOI:** 10.3389/fbioe.2025.1579062

**Published:** 2025-06-06

**Authors:** Youngshim Choi, Yinyan Ma, Samson Tom, Alla Danilkovitch, Liqing Yu

**Affiliations:** ^1^ Division of Endocrinology, Diabetes and Nutrition, Department of Medicine, University of Maryland School of Medicine, Baltimore, MD, United States; ^2^ Britecyte, Inc., Frederick, MD, United States

**Keywords:** adipose tissue, adipose stem cells, tissue engineering, MASLD, fibrosis, inflammation

## Abstract

**Introduction:**

Metabolic dysfunction-associated steatotic liver disease (MASLD) and its progressive stage metabolic dysfunction-associated steatohepatitis (MASH) represent a leading cause of liver-related morbidity and mortality in the U.S. and worldwide. Given the high prevalence and rapid growth of MASLD, the economic and health burden, and MASH-associated morbidity and mortality, there is an unmet medical need for therapies that can stop, slow, or reverse the progression of MASLD. Adipose tissue plays a key role in metabolic health and MASLD pathogenesis through its regulation of energy metabolism and endocrine function. Metabolic dysfunction is often associated with a chronic state of low-grade inflammation in the body including adipose tissue.

**Methods:**

In this study, we tested an engineered adipose formulation (AF) composed of a combination of culture-expanded adipose stromal vascular fraction (SVF) cells and adipose tissue particulates in the treatment of MASLD. Human, rat, and mouse AFs (hAF, rAF, and mAF) showed anti-inflammatory activity, which was mediated predominantly by soluble factors present in the adipose particulate. *In vivo* effects of AFs were evaluated in two rodent models of MASLD: i) obese Zucker rats fed a high-fat, high-cholesterol, and high-fructose diet that mainly manifest hepatic steatosis; and ii) liver-specific CGI-58 knockout mice (LivKO) on a Western diet that display MASH pathologies.

**Results:**

Subcutaneous implantation of hAF and rAF in obese Zucker rats significantly reduced hepatic triglycerides. In LivKO mice, mAF reduced hepatic inflammation and fibrosis, though not steatosis, as evidenced by significant decreases in hepatic M1 macrophages and mRNAs for proinflammatory and fibrogenic genes. Immunogenicity testing demonstrated that allogeneic rAF did not induce an immune response, whereas, as anticipated, xenogeneic hAF in rats triggered anti-hAF antibody formation. Despite an immune response against xenogeneic hAF, treatment of rats with hAF ameliorated hepatic steatosis.

**Discussion:**

AF has the potential to treat MASH. Future studies focused on the optimization of AF composition, optimal dose and treatment regimen are warranted.

## Introduction

Metabolic dysfunction-associated steatotic liver disease (MASLD) and its progressive stage, metabolic dysfunction-associated steatohepatitis (MASH) (Rinella et al., 2023), are the most common chronic metabolic liver diseases and are the leading causes of liver-related morbidity and mortality worldwide (Teng et al., 2023; Younossi et al., 2023). In the U.S. alone, approximately 30% and 5% of the population are affected by MASLD and MASH, respectively (Cotter and Rinella, 2020). Approximately 20% of people with MASH progress to cirrhosis, resulting in liver failure and the need for liver transplantation (Younossi, 2019). According to the 2022 annual data report from the Organ Procurement and Transplantation Network and the Scientific Registry of Transplant Recipients (OPTN/SRTR), MASH is the second leading diagnosis for liver transplants (Kwong et al., 2024), and the demand for liver transplants among MASH patients is rapidly increasing (Younossi et al., 2021). An alarming trend shows an increase in MASH among MASLD patients, predicting that liver-related mortality will more than double in the coming years (Estes et al., 2018). In the MASH population, 370,000 total and 28,200 liver-related deaths were recorded in 2015, with projections of 716,800 total deaths and 78,300 liver-related deaths expected by 2030 (Estes et al., 2018). The Global Assessment of the Impact of MASH (GAIN) study indicated a substantial burden on health services and individuals: it is estimated that with a MASH prevalence of 5.3%, the total annual national economic burden of MASH in the U.S. exceeds $80 billion (O'Hara et al., 2020). Given the growing prevalence, the economic and health burden of MASLD, and their associated morbidity and mortality, there is an urgent unmet medical need for therapies that can halt, slow the progression of, or reverse MASH. Over the past 40 years, pharmaceutical companies’ efforts to develop drugs for MASH have not been successful, with only Resmetirom, a thyroid hormone receptor beta (THR-β) agonist, approved in 2024 (Kokkorakis et al., 2024). Considering the many failed attempts, the approval of Resmetirom is significant; however, only 25%–30% of MASH patients respond to the treatment (Author anonymous, 2024). MASH is a complex disease characterized by hepatic steatosis, inflammation, and fibrosis. The etiology of MASH is multifactorial, including dysregulated substrate metabolism and immunological responses due to nutritional imbalance and changes in genetic and epigenetic landscapes (Author anonymous, 2024). Therapies that address multiple pathologies of MASH remain an unmet need.

The historical view of adipose tissue as an organ that passively stores excess calories has evolved. Adipose tissue is now recognized as the largest endocrine organ in the body, regulating glucose and lipid metabolism, inflammation, and orchestrating tissue regeneration (Kershaw and Flier, 2004; Harvey et al., 2020). Lipotoxic liver injury resulting from adipose tissue dysfunction can lead to MASH (Folestad and Falkevall, 2024). Studies have shown that transplanting healthy human or mouse adipose tissue into obese mice restores disrupted metabolism, resulting in reductions in weight, insulin resistance, and liver steatosis (Klebanov et al., 2005; Ablamunits et al., 2012). These observations suggest that adipose tissue, through its multiple biological activities, can address the complexity of MASH. Current expert opinion attributes the therapeutic properties of adipose tissue to resident mesenchymal stem cells (MSCs) found in the stromal vascular fraction (SVF) of the tissue (Mizuno, 2009; Bianchi et al., 2013; Bora and Majumdar, 2017). Consistently, animal studies have demonstrated that injecting MSCs into rodents on a high-fat diet can improve metabolism and liver function (Domingues et al., 2019; Li et al., 2019). However, adipose tissue formulations are more effective than isolated adipose SVF cells (Kokai et al., 2022), highlighting the importance of the tissue microenvironment. The extracellular matrix of adipose tissue likely provides a favorable environment to support SVF cell attachment, survival, differentiation, and function. Understanding the functionality of adipose tissue in the body has led to its clinical applications rapidly, expanding from traditional applications as structural grafts for reconstructing adipose defects to treating inflammatory and metabolic diseases (Dige et al., 2019; Nilforoushzadeh et al., 2019; Xu et al., 2019; Ersozlu and Gultekin, 2020; Onorato et al., 2024). However, because of the immunogenicity of allogeneic adipose, its clinical applications are limited to autologous tissues only. Overcoming the immunogenicity of allogeneic adipose tissue is crucial for developing off-the-shelf adipose therapies for many diseases with unmet medical needs.

Recognizing the therapeutic potential of adipose tissue, a novel method for processing adipose tissue has been developed. Adipose tissue is mechanically converted into micro-particulates consisting of extracellular matrix, devitalized adipocytes, and other cell types. The method preserves the structure and function of native adipose tissue while simultaneously eliminating immunogenic components, including blood cells, allowing for the allogeneic use of the tissue (Regulski et al., 2024). Based on the known therapeutic effects of adipose tissue and MSCs, we engineered an adipose formulation (AF) composed of a combination of adipose particulates and MSCs derived from culture-expanded adipose stromal vascular fraction (SVF) cells. Adipose SVF was chosen because it is a rich source of MSCs. After one passage in culture, almost all adipose SVF cells start to express MSC markers and demonstrate functional properties of MSCs. This biocompatible “off-the-shelf” AF has the potential to address the complexity of MASH etiologies and pathologies by interrupting multiple pathological pathways that drive the disease progression. Other advantages of the AF include its on-demand availability, prolonged storage, easy implantation via a minimally invasive injection, and no need for patients to do Human Leukocyte Antigen (HLA) matching or use immunosuppressants. The developed allogeneic adipose tissue technology allows for the efficient use of donated human tissues by transforming discarded adipose tissue from medical waste into therapeutic products. A large supply of adipose tissue makes it feasible to scale the manufacturing process to meet the anticipated high market demand in the future. In this study, we characterized our AF and its tissue and cell components, and evaluated the safety and therapeutic effects of AF in two animal models of MASLD that represented the early and late stages of MASLD.

## Methods

### Statement of ethics of human adipose tissue procurement and animal care and use

Human adipose tissues were provided by the National Disease Research Interchange (NDRI, Philadelphia, PA) and ZenBio, Inc. (Durham, NC) from eligible donors according to their approved protocols, with written informed consent obtained. NDRI and ZenBio also provided the tissue procurement and ethics statements. All animal procedures were approved by the Institutional Animal Care and Use Committee (IACUC) of the Noble Life Sciences and the University of Maryland, Baltimore. All animal procedures were performed in accordance with the U.S. National Institute of Health guidelines, including “Principles for Use of Animals” and “Guide for the Care and Use of Laboratory Animals”. All procedures were conducted in compliance with the current versions of the following guidelines: 1) Animal Welfare Act Regulations (9 CFR); 2) U.S. Public Health Service Office of Laboratory Animal Welfare Policy on Humane Care and Use of Laboratory Animals; 3) Guide for the Care and Use of Laboratory Animals (Institute of Laboratory Animal Resources, Commission on Life Sciences, National Research Council, 1996); and 4) the Association for Assessment and Accreditation of Laboratory Animal Care International (AAALACi).

### Human adipose SVF cell preparation and characterization

#### Adipose stromal vascular fraction (SVF) cell isolation, culturing, and cryopreservation

SVF cells were isolated from adipose tissue by enzymatic digestion. Approximately 5 mL (∼4.5 g) of 3–5 mm adipose tissue were placed in a tube containing 0.25 mL of 2% collagenase type 2 solution (Worthington, Lakewood, NJ) in 15 mL of DMEM with 10% FBS. The tube was incubated at 37°C on a rotator for 30–45 min. Then, the digested tissue was filtered through a 300 mm nylon mesh strainer and centrifuged for 5 min at 1,200 rpm at room temperature (RT). The cell pellet containing SVF cells was collected and plated in tissue culture flasks in RoosterNourish™ MSC-XF medium (RoosterBio, Frederick, MD). SVF cells were seeded in a 75 cm^2^ flask at a density of 2,000–3,000 cells/cm^2^ and cultured for 6 days in RoosterNourish™ MSC-XF medium. After 6 days, the cells typically reached 75%–80% confluency, and were harvested using TrypLE (Life Technologies, Carlsbad, CA), then centrifuged, counted, and re-plated in new 75 cm^2^ flasks at 3,000 cells/cm^2^ for further expansion or cryopreservation. SVF cells were mixed with CryoStor CS10 freezing medium (Life Technologies, Carlsbad, CA), transferred to cryovials, and stored at −80°C prior to use.

#### Human adipose SVF cell phenotyping by flow cytometry

Cryopreserved human adipose SVF cells at the second passage (P2) were thawed, counted, and stained with the properly conjugated antibodies CD34, CD45, CD73, CD90, and CD105 (BioLegend^®^, San Diego, CA). Properly conjugated isotype-matched antibodies were used as a negative control (Biolegend^®^, San Diego, CA). The analysis was performed either by FACS-Calibur or FACSCanto (BD Bioscience, Piscataway, NJ).

#### Adipogenic differentiation of human adipose SVF cells

For adipogenic differentiation, SVF cells at P2 were thawed, counted, seeded in 24-well plates (1 × 10^4^ cells per well), and cultured in the RoosterNourish™ MSC-XF medium (RoosterBio, Frederick, MD) to reach confluency. The medium was then replaced with the adipogenic differentiation medium (Life Technologies, Carlsbad, CA). The adipogenic medium was changed every 3–4 days, and cells were maintained in the culture for 2 weeks. Cells cultured in DMEM containing low FBS served as a control. Adipogenic differentiation was detected by Oil Red O lipid staining (Sigma, St. Louis, MO).

#### Response of human adipose SVF cells to hypoxia

To evaluate response to hypoxia, human adipose SVF cells at P2 were thawed, counted, seeded in 24-well plates (1 × 10^4^ cells per well) and cultured in RoosterNourish™ MSC-XF medium (RoosterBio, Frederick, MD) for 48 h in a hypoxic chamber (1% O_2_). Cells cultured in normoxic conditions (20% O_2_) served as a control. After 48 h, culture supernatants were collected, and human VEGF was measured by ELISA (R&D Systems, Minneapolis, MN).

### Adipose particulate preparation and characterization

#### Adipose particulate processing and cryopreservation

Human adipose tissues (from lipoaspirate or cadaveric donors) were cut into small (3–5 mm) pieces using scissors (only for cadaveric donors; adipose tissues collected via lipoaspiration were already in ∼3 mm pieces and did not require cutting with scissors), which were then further homogenized into a ∼1–2 mm tissue particle size using a knife. After that, the tissue was filtered and washed with saline through 1 mm and 0.71 mm sieves. The free oil released from cut adipocytes was removed by centrifugation, and the adipose tissue particulate was saturated with a cryopreservation solution containing 0.5 M trehalose (Sigma, St Louis, MO) and 2.5% human serum albumin (HSA) (OctaPharma, Hoboken, NJ) and packaged in 2 mL cryovials or in syringes. The processed adipose tissue resulted in a tissue particulate that can be implanted using a 20–21-gauge (G) needle. Rodent adipose tissues were collected from Sprague Dawley (SD) rats and C57Bl/6 mice at Noble Life Sciences. The tissue processing and cryopreservation mirrored the method developed for the human adipose tissue. The human, rat, and mouse adipose tissue particulates were stored frozen in a −80°C freezer prior to use.

#### Histological evaluation of human adipose tissue (hAT) and particulate (hAP)

Histological processing and H&E staining of adipose samples were performed at Histoserv, Inc. (Germantown, MD).

#### Evaluation of hAP immunogenicity in LPS-induced TNF-α secretion assay *in vitro*

The absence of immunogenic components in hAP was confirmed through a Lipopolysaccharide (LPS)-induced TNF-α secretion assay. Human adipose tissue before processing (starting material) and post-thaw processed hAP from the same donor were cultured in a 24-well plate in RoosterNourish™ MSC-XF medium (RoosterBio, Frederick, MD) in the presence of 5 μg/mL bacterial LPS (Sigma, St. Louis, MO, United States) for 48 h at 37°C. Samples without LPS served as controls. After 48 h, the culture supernatants were collected and tested for detecting human TNF-α using a human TNF-α DuoSet kit (R&D Systems, Minneapolis, MN) according to the manufacturer’s protocol.

#### Growth factor and cytokine profile of hAP and human adipose SVF cells

The presence of growth factors and cytokines in human adipose particulate (hAP) extracts (hAPEs) or culture supernatants derived from human adipose SVF cells was evaluated in a multiplex Luminex assay using a ProcartaPlex^™^ Human Angiogenesis Panel, 18-plex (Life Technologies, Carlsbad, CA) and a ProcartaPlex^™^ Human Inflammatory Panel, 6-plex, custom (Life Technologies, Carlsbad, CA) or using a Human Adiponectin/Acrp30 DuoSet ELISA kit (R&D Systems, Minneapolis, MN) according to the manufacturer’s protocols. Culture supernatants for testing were collected, and APEs were prepared by mixing 1:1 (V/V) tissue and RPMI or DMEM without FBS in Eppendorf tubes. Tubes were then vortexed and incubated on a rotator for 2 h at RT and centrifuged at 13,000 rpm for 10 min at RT. APEs were transferred into new Eppendorf tubes. All tubes with APEs were stored at −80°C prior to testing. Culture supernatants derived from human adipose SVF cells were collected 72 h after plating.

### Fabrication and characterization of adipose formulations (AFs)

#### Fabrication of adipose formulations (AFs)

Human adipose formulation (hAF) is comprised of two components from the same donor: culture-expanded human adipose-derived SVF cells and adipose particulate (AP). Culture-expanded human SVF cells harvested from tissue culture flasks or thawed previously frozen SVF cells from a vial were mixed at 1 million cells per 1 mL AP for cell seeding. The fabricated hAFs were tested on the same day or cryopreserved in a solution containing trehalose, dimethyl sulfoxide (DMSO), and HSA and stored at −80^0^C prior to use. For the fabrication of rat (rAF) and mouse (mAF) adipose formulations, the same procedures for the fabrication of hAF described above were performed by using rAF and mAF mixed with rAP and mAP, respectively.

#### Secretion of human VEGF and adiponectin by hAF and its components, hAP, and human adipose SVF cells

Human VEGF and adiponectin were tested in culture supernatants derived from hAF, hAP, and human adipose SVF cells by using human VEGF DuoSet and Human Adiponectin/Acrp30 DuoSet ELISA kits (R&D Systems, Minneapolis, MN) according to the manufacturer’s protocols.

#### Anti-inflammatory activity of hAF and its components, hAP, and human adipose SVF cells

The anti-inflammatory activity of hAF, hAP, and human adipose SVF cells was evaluated by detecting the inhibition of inflammatory cytokine secretion from LPS-stimulated THP-1 cells. THP-1 cells were seeded into 24-well plates (1 million cells/well) in RPMI 1640 medium with 5% FBS and treated with 0.5 μg/mL LPS combination with 0.1 mL of hAF or 0.1 mL of hAP or human adipose SVF cells (0.1 million cells/well) for 48 h. THP-1 cells alone in the medium (unstimulated cells) and treated with LPS only served as controls. After 48 h, tissue culture supernatants were collected and centrifuged; then, supernatants were transferred to new tubes. The levels of inflammatory cytokine TNF-α were tested by using a Human TNF-α DuoSet ELISA kit (R&D Systems, Minneapolis, MN) according to the manufacturer’s protocols.

#### Anti-inflammatory activity of human, rat, and mouse adipose particulates (hAP, rAP, mAP) and their extracts (hAPE, rAPE, mAPE)

Human, rat, and mouse APs were prepared as described in the “Adipose Particulate Processing and Cryopreservation” section. Human, rat, and mouse APEs were prepared as described in the “Growth Factor & Cytokine Profile of hAP And Human Adipose SVF Cells” section. The anti-inflammatory activity of APs and APEs was evaluated by detecting the inhibition of inflammatory cytokine secretion from LPS-stimulated THP-1 cells as described in the “Anti-inflammatory Activity of hAF and its Components, hAP, and Human Adipose SVF Cells” section. hAPs were tested at 0.05, 0.1, and 0.2 mL concentrations, and hAPEs were tested at 25% and 50% concentrations. rAP and mAP were tested at a 0.1 mL concentration, and rAPE and mAPE were tested at a 50% concentration.

### Testing of adipose formulations in MASLD and MASH with fibrosis rodent models

#### Rodents and diets

Zucker rats represent a well-characterized model of MASLD. Eight to ten-week-old male obese (fa/fa) Zucker rats (Envigo, Madison, WI) were fed the high fat, cholesterol, and fructose (HFC-F) diet (Special Diet Quote # TD.200631, Lard & Beef Tallow Diet, Envigo, Madison, WI) *ad libitum* for 4 weeks. The HFC-F diet consists of 25% fat (in the form of lard and beef tallow), 5% cholesterol, and 35% fructose by weight. After 4 weeks, the rats were treated with AFs, followed by an additional 4 weeks of feeding with the HFC-F diet prior to necropsy. CGI-58 LivKO mice were generated as described previously (Guo et al., 2013). Homozygous CGI58-floxed mice with albumin promoter-driven cre transgene (LivKO) and ALB-control mice were fed *ad libitum* a standard chow diet (LabDiet) from weaning to 6 weeks of age. Then, the mice were fed *ad libitum* a Western diet (D12079b, Research Diets, Inc.) for 8 weeks before treatment with AFs, followed by an additional 4 weeks of feeding with a Western diet prior to necropsy. All animals were housed in specific pathogen-free animal facilities at 22°C with a 12 h light/dark cycle with lights on from 6 a.m. to 6 p.m.

#### Testing of AFs in a HFC-F diet-induced obese Zucker rat MASLD model

In this study, 21 obese fa/fa Zucker male rats (n = 6 per group and n = 3 baseline control) at the age of 8–10 weeks were used. The baseline control rats (n = 3) were sacrificed at the beginning of the study before HFC-F diet feeding. Livers and blood were collected; the livers and mice were weighed to calculate the liver/body weight ratio. The remaining 18 rats were fed the HFC-F diet for 4 weeks and then randomly divided into 3 different treatment groups (n = 6), in which mice received a 2 mL subcutaneous injection in 4 points (0.5 mL/point) with rat PBS (control), hAP, or rAP (derived from SD rats). After the treatment, rats continued to stay on the HFC-F diet for an additional 4 weeks prior to euthanasia. All animals were weighed weekly for the duration of the study. At the end of the study, gross examination was performed, and blood serum and liver samples were collected. The injection sites were examined, and the adipose formulations were photographed and dissected. Samples collected for histology were fixed in 10% Neutral Buffered Formalin. All other samples were stored frozen prior to testing. Endpoints included daily animal health, weekly body weight (BW), blood serum chemistry, gross evaluation, liver histology and triglycerides, and immunogenicity of rats injected with hAF or rAF formulations.

#### Immunogenicity of hAF and rAF *in vivo*

Immunogenicity of hAF and rAF *in vivo* was investigated by the detection of anti-hAP and anti-allogeneic Sprague Dawley (SD)-derived rAF antibodies in blood serum of obese Zucker rats. Blood serum samples were collected 4 weeks after treatment and stored frozen in aliquots before testing. Detection of anti-hAF and anti-allogeneic rAF antibodies in rat serum was done by ELISA using hAF and rAF extracts in PBS. The necessary reagents for the ELISA assay were contained in the DuoSet ELISA Ancillary Reagent Kit 2 (R&D Systems, Minneapolis, MN). A 96-well ELISA plate was coated with 4 μg/mL of the hAF or rAF extract. After plate blocking, rat serum samples were titrated using 2-fold dilutions ranging from 1:100 (1%) to 6,400 (0.016%). The plate containing the titrated rat serum was then incubated for 2 h at RT. The presence of antibodies was detected using goat anti-rat immunoglobulin G (IgG) antibodies conjugated with horseradish peroxidase (HRP) (1:1,000 dilution, R&D Systems, Minneapolis, MN), followed by the tetramethyl benzidine (TMB) substrate.

#### Rat hepatic triglyceride content and liver histology

Hepatic triglyceride content in rat livers was quantified using a commercially available kit (Cayman, Ann Arbor, MI) according to the manufacturer’s protocol, except using larger liver samples (∼2 g). Histological staining and analyses were performed at Histoserv (Germantown, MD).

#### Testing of AFs in Western Diet-induced MASH and a hepatic fibrosis model using liver CGI-58 knockout (LivKO) mice

Liver CGI-58 knockout (LivKO) mice were generated as described by Guo et al. (2013) LivKO and ALB-control mice at 6 weeks of age were fed the Western Diet for 8 weeks and then randomly divided into 3 different treatment groups (n = 8–9), in which mice received a 0.5 mL subcutaneous injection in 4 points (0.125 mL/point) with mouse PBS (control), hAP, or mAP (derived from C57Bl/6 mice). After the treatment, mice continued to stay on the Western diet for an additional 4 weeks prior to euthanasia. At the end of the study, gross examination was performed, and blood serum and liver samples were collected. The injection sites were examined, and the adipose formulations were photographed and dissected. Samples collected for histology were fixed in 10% Neutral Buffered Formalin. All other samples were stored frozen prior to testing. Endpoints included daily animal health, weekly BW, gross evaluation, liver and adipose tissue weight, liver histopathology, hepatic triglycerides, inflammatory markers, liver enzymes, leptin, and adiponectin levels in blood serum of mice injected with hAF or mAF formulations. In addition, M1 and M2 macrophages in the liver tissues were evaluated using immunohistochemistry (IHC).

#### Mouse blood parameters

Serum alanine transaminase (ALT) and aspartate transaminase (AST) activities were determined by using commercial kits (Point Scientific, Inc., Canton, MI) according to the manufacturer’s instructions. Serum adiponectin and leptin concentrations were measured using a Mouse Adiponectin/Acrp30 ELISA kit and a Mouse Leptin DuoSet ELISA kit, respectively (R&D Systems, Minneapolis, MN).

#### Lipid extraction and TG level in liver

Hepatic lipids were extracted from 100 mg of liver tissue in a chloroform:methanol (2:1) solution. The sample tube was incubated overnight at RT and then placed into a heater (60°C) for 1 h. After adding 1.5 mL of 0.1 M NaCl to the tube, centrifugation was performed at 1,000 rpm for 10 min at RT. The lower organic phase was transferred to a new tube, and then the tube was put into the air-dry module until the organic solvent completely evaporated. The sample tube was placed into a heater (70°C) for 1 h after adding 200 μL of 3 M potassium hydroxide (KOH) in 65% ethanol (EtOH). Hepatic TG levels were measured with a Triglyceride Colorimetric Assay Kit (Cayman, Ann Arbor, MI) according to the manufacturer’s instructions. The values were normalized by wet liver tissue weight or protein amount.

#### Hepatic oxidative stress

Hepatic levels of the lipid peroxidation product malondialdehyde (MDA) were measured as thiobarbituric acid reactive substrates (TBARS) using a TBARS Assay Kit (R&D Systems, Minneapolis, MN) following the manufacturer’s instructions.

#### Mouse liver histological analysis

Formalin-fixed liver tissues were paraffin-embedded, and sections were stained with either hematoxylin and eosin (H&E) or Picrosirius red. The expression of CD68 and CD206 in the liver was evaluated by immunohistochemical staining using primary antibodies against mouse CD68 (1:4,000 dilution, Abcam, Waltham, MA) or mouse CD206 (1:1,600 dilution, Cell Signaling Technology^®^, Danvers, MA). All formalin-fixed liver tissue processing and staining were performed at Histoserv (Germantown, MD). Liver fibrosis assessment was conducted by an experienced, blinded investigator using a scale from 0 (absent) to 4 (1, perisinusoidal/pericellular fibrosis; 2, periportal fibrosis; 3, bridging fibrosis; 4, cirrhosis).

#### Hepatic RNA extraction and quantitative real-time PCR

Total RNA was extracted from liver samples using TRIzol reagent (Invitrogen, Carlsbad, CA) and reverse transcribed using TaqMan™ Reverse Transcription Reagents (Invitrogen, Carlsbad, CA). Real-time PCR was performed using Applied Biosystems’ Power SYBR™ Green PCR Master Mix on a QuantStudio™ 6 Pro Real-Time PCR System (Applied Biosystems, Foster City, CA) according to the manufacturer’s instructions. The relative mRNA expression level for each gene was calculated using the 2^(−DDCt)^ method and normalized to GAPDH.

#### Statistical methods

All statistics were calculated using GraphPad Prism 10.4.1 and Microsoft Excel. Student’s *t*-test was used for statistical analysis, and p < 0.05 was considered significant.

## Results

### Study design

**FIGURE 1:**
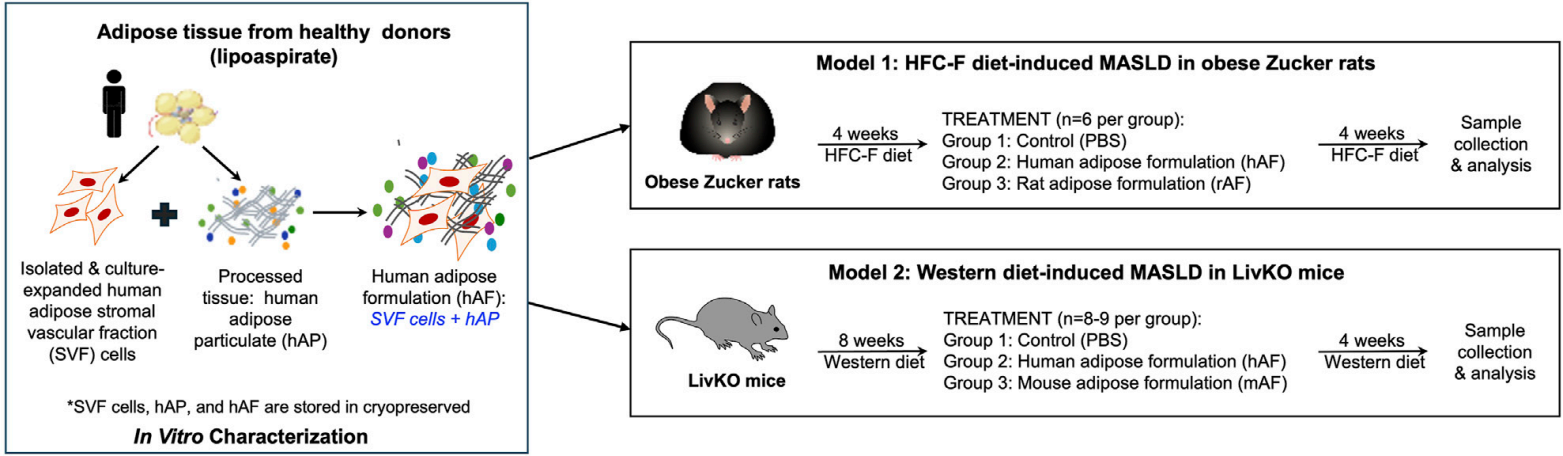
Schematic presentation of the study design.

This study describes the results of *in vitro* and *in vivo* testing of a human adipose formulation (hAF), which is designed for the treatment of MASLD/MASH. The study contained 3 distinct parts: 1. Fabrication and *in vitro* characterization of hAF and its components: human adipose particulate (hAP) and culture expanded adipose-derived stromal vascular fraction (SVF) cells; 2. Evaluation of the therapeutic effects of hAF in obese Zucker rats in a MASLD model; and 3. Evaluation of the therapeutic effects of hAF in severe MASH with hepatic fibrosis in CGI-58 liver-specific knockout (LivKO) mice. As it was anticipated that xenogeneic hAF would trigger an immune response in immune-competent animals, allogeneic rat (rAF) and mouse (mAF) formulations, which mimic a future proposed clinical use of hAF, were included in the rat and mouse models, respectively. The composition and fabrication of rAF and mAF mirrored the composition and fabrication of hAF. The study design is presented in Figure 1.

### *In vitro* characterization of hAF and its components

#### Characterization of culture-expanded adipose-derived SVF cells

**FIGURE 2:**
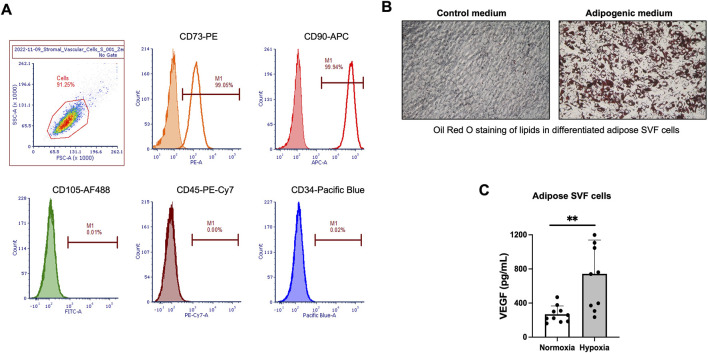
*In vitro* characterization of culture-expanded human adipose-derived stromal vascular fraction (SVF) cells. **(A)** Flow cytometry histograms for selected mesenchymal and hematopoietic cell markers in human adipose-derived SVF cells at passage 2 (P2). The percentage of cells staining positive is indicated in the right corner of each panel. The closed histograms indicate cell staining with isotype control antibodies. **(B)** Representative images of Oil Red O staining in differentiated SVF cells incubated in adipogenic medium. **(C)** Secretion of human vascular endothelial growth factor (hVEGF) in culture supernatants from human SVF cells after 48 h exposure to hypoxia. Normoxia served as a control. Data are presented as mean ± SD for 10 donors. *P < 0.05 by Student’s t-test.

SVF cells were isolated from subcutaneous adipose lipoaspirates derived from 10 donors using enzymatic digestion of the tissues. The isolated SVF cells were rapidly grown in the RoosterNourish™ MSC-XF medium designed to support the growth of mesenchymal stromal cells. The adherent SVF cells had spindle-shaped mesenchymal cell- and fibroblast-like morphology. The SVF cells at passage 2 (P2) appeared to consist of a more homogenous cell population. Cells were cryopreserved, and characterization analyses were performed on the cryopreserved SVF cells at P2. Cell phenotype, adipogenic differentiation, and response to hypoxia were key tests to characterize the SVF cells. Flow cytometry evaluated cell phenotype, and SVF cells at P2 were positive for mesenchymal markers CD73 and CD90 while being negative for CD105. The SVF cells were negative for hematopoietic markers CD45 and CD34 (Figure 2A). The SVF cell population was uniformly homogeneous based on the expression of these 5 cell markers: more than 98% of SVF cells were positive for CD73 and CD90 expression, and less than 0.5% of SVF cells were CD105, CD45, and CD34 positive (Figure 2A). This cell phenotype was observed across all tested donors. Next, the SVF cells from all donors underwent adipogenic differentiation (Figure 2B) and responded to hypoxia by significantly upregulating VEGF secretion (Figure 2C).

#### Characterization of hAP

**FIGURE 3:**
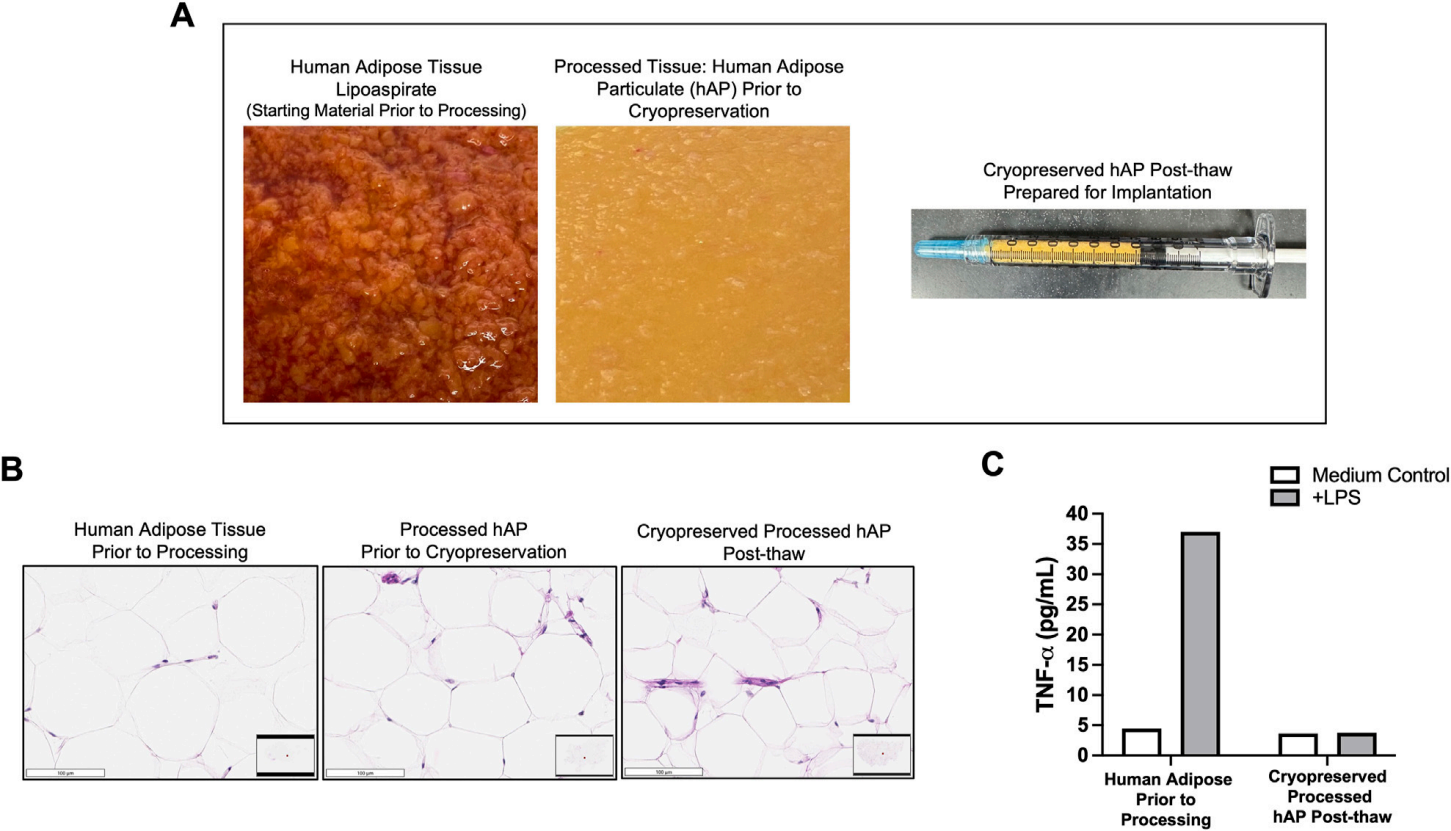
*In vitro* characterization of human adipose particulate (hAP). **(A)** Representative images of human adipose tissue collected through lipoaspiration (left), hAP after processing (middle), and a syringe filled with hAP that was prepared for implantation into animals (right). **(B)** Microphotographs of H&E-stained tissue sections from human adipose tissue before processing, processed hAP before cryopreservation, and cryopreserved hAP after thawing. Scale bars: 100 μm. **(C)** TNF-α secretion in culture supernatants from human adipose before processing and cryopreserved processed hAP after thawing following LPS stimulation. Results for one representative experiment are displayed.

hAP was generated using a developed processing method for human adipose tissue. The development of the adipose tissue processing was focused on preserving the structure of the native adipose tissue, removing immunogenic components, and converting the procured donor’s adipose tissue into a particulate that can be implanted via a minimally invasive injection. The tissue was cut with a blade, followed by multiple washing steps, and adipose pieces were filtered through 1 mm and 0.7 mm nylon filters. At the end of processing, the resulting hAP was yellow in color without any visible contamination with blood or non-adipose tissue and could be implanted using 20–21G hypodermic needles (Figure 3A). For long-term storage, hAP was cryopreserved in a trehalose-HSA-containing solution in cryovials or hypodermic syringes. Characterization of hAP was performed after thawing. Histologic analysis confirmed that hAP retained the structure of the native adipose tissue (Figure 3B). *In vitro* immunogenicity of cryopreserved hAP was evaluated in the LPS challenge assay. Human adipose tissue prior to processing released a significant amount of TNF-α after LPS stimulation, indicating the presence of immunogenic components in the tissue. In contrast, hAP did not release TNF-α in response to LPS stimulation (Figure 3C).

**FIGURE 4:**
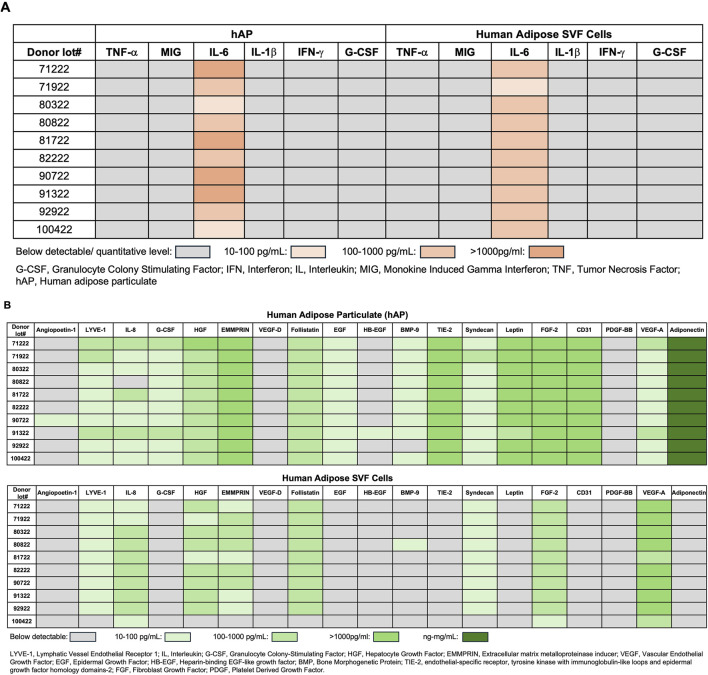
Growth factor and cytokine profile of human adipose particulate (hAP) and SVF cells. **(A)** Detection of inflammatory cytokines; **(B)** angiogenic growth factors, leptin, and adiponectin in hAP extracts (hAPEs) or culture supernatants derived from human adipose SVF cells of 10 donors. Levels of cytokines and growth factors are presented as a heat map.

Evaluation of growth factors and cytokines released by hAP and SVF cells revealed that among 6 inflammatory cytokines, only IL-6 levels were detectable in hAP and SVF cells (Figure 4A). Although VEGF levels produced by hAP were significantly lower when compared with the VEGF levels secreted by SVF cells from the same donors, the results demonstrated that both hAP and SVF cells secreted angiogenic factors (Figure 4B). Leptin and adiponectin, hormones secreted by adipose tissue, were detected only in hAP (Figure 4B).

#### Characterization of hAF

**FIGURE 5:**
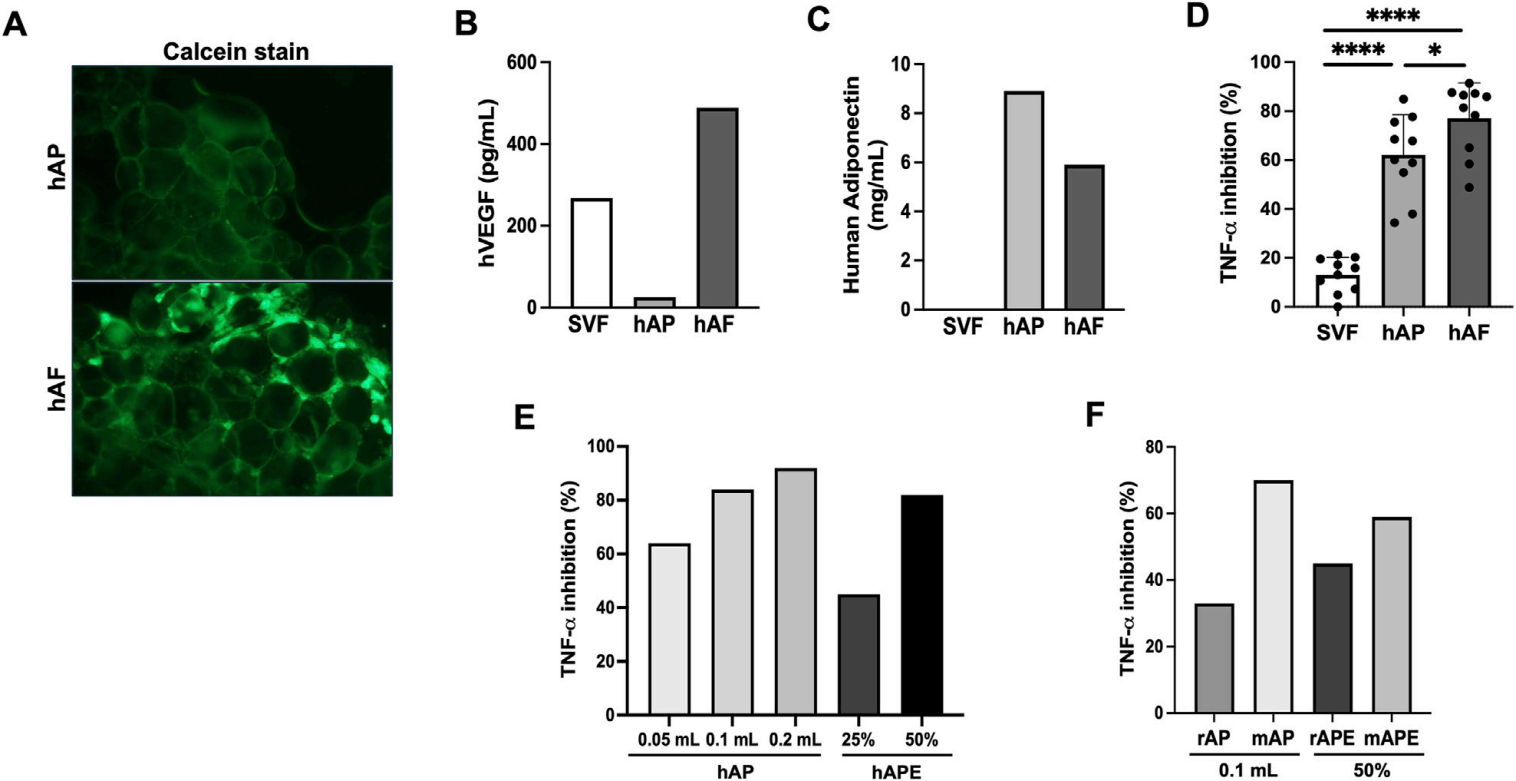
*In vitro* characterization of human-engineered adipose formulation (hAF) comprised of hAP seeded with SVF cells. **(A)** Fluorescent images of Calcein stained hAP (top) and hAF (SVF cells + hAP) (bottom) post-thaw. **(B)** hVEGF secretion by hAF or its components, hAP and SVF cells. Results from a representative donor are shown. **(C)** Human adiponectin secretion by hAF or its components, hAP and SVF cells. Results from a representative donor are shown. **(D)** Anti-inflammatory activity of hAF and its components was evaluated by inhibition of TNF-α secretion in LPS-stimulated THP-1 cells. Data are presented as mean ± SD for 10 donors. **(E)** Anti-inflammatory activity of hAP and hAP extracts (APEs) evaluated by inhibition of TNF-α secretion in LPS-stimulated THP-1 cells in a dose-dependent manner. Results from a representative donor are shown. **(F)** Anti-inflammatory activity of rat (rAP) and mouse (mAP) adipose particulate and their AP extracts (rAPE and mAPE) evaluated by inhibition of TNF-α secretion in LPS-stimulated THP-1 cells. Results from a representative donor are shown. *P < 0.05, **P < 0.01 by Student’s t-test.

For the fabrication of hAF, SVF cells were mixed at 1 million cells per 1 mL hAP. After overnight incubation, unattached cells to hAP were removed, and hAP with attached SVF cells were cryopreserved in a trehalose-HSA-containing solution. Fabricated hAF was characterized after thawing. The presence of viable cells in hAP was observed by fluorescent microscopy after staining hAF with Calcein. Figure 5A shows that hAP had no viable cells (Top image), and hAF contained SVF cells attached to hAP (Bottom image). Detection of VEGF and adiponectin secreted by hAF in the tissue culture medium confirmed the results observed in the profile of hAP and SVF cells (Figure 4B): SVF cells are the source of VEGF (Figure 5B), and hAP is the source of adiponectin (Figure 5C) in the hAF formulation. As liver inflammation is a hallmark of MASH, hAF and its components were tested for anti-inflammatory activity using the THP-1 cell line, a model for human monocytes/macrophages. Previous studies have shown that THP-1 cells, when stimulated with LPS, exhibit very similar gene transcription and cytokine secretion profiles compared to PBMC-derived macrophages (Sharif et al., 2007; Scheildberger et al., 2013). Also, THP-1 cells provide a consistent cell population, unlike the variability seen between different human donors, allowing for reliable and comparable results to test drug candidates for anti-inflammatory activity. Results demonstrated that hAF has strong anti-inflammatory activity: it significantly inhibited the secretion of TNF-α in LPS-stimulated THP-1 cells, which is mediated by the hAP component, whereas SVF cells showed negligible inhibition of TNF-α (Figure 5D). The results with hAP and hAP-derived extracts (hAPEs) demonstrated that the anti-inflammatory activity of hAP is dose-dependent and mediated by soluble factors present in hAP (Figure 5E). Similar to findings with hAF, rat and mouse APs also showed dose-dependent anti-inflammatory activity mediated by soluble factors (Figure 5F).

### Evaluation of hAF and rAF in a MASLD model using Obese Zucker rats

**FIGURE 6:**
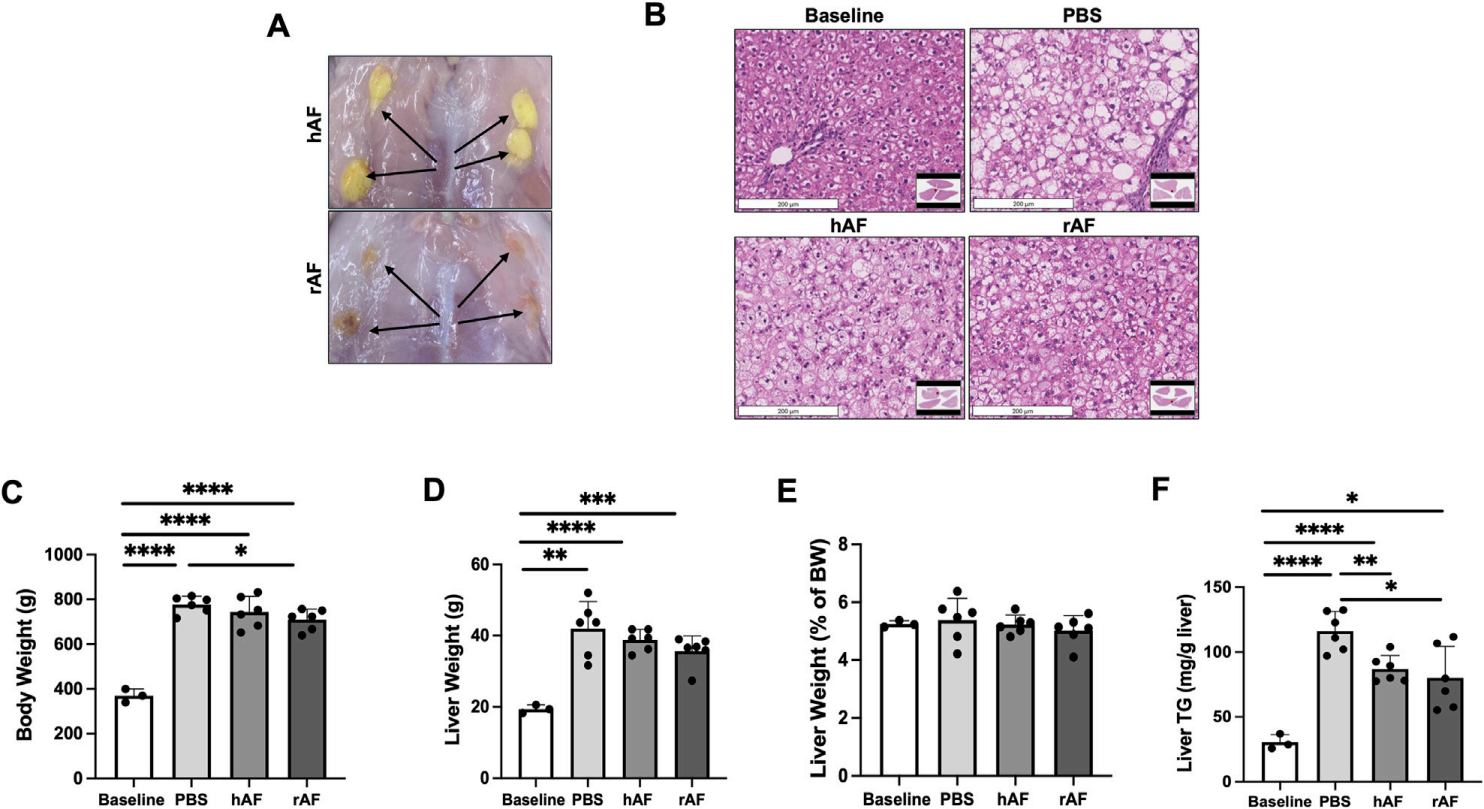
Effects of hAF or rAF treatment on MASLD in obese Zucker rats. **(A)** Images of hAF (top) and rAF (bottom) in rats 4 weeks after SC implantation. Arrows point to hAF and rAF. **(B)** Representative images of H&E-stained liver tissue in rats. Scale bars: 200 μm. **(C)** Body weights, **(D)** liver weights, **(E)** liver triglycerides (TG), and **(F)** liver/body weight ratio in rats at the end of the study. Baseline: rats prior to HFC-F diet. Data are presented as mean ± SD. n = 6 for treatment group, and n = 3 for baseline group. *P < 0.05 by Student’s t-test.

This animal study aimed to evaluate the therapeutic potential of hAF and rAF to treat MASLD using obese fa/fa Zucker rats. Male rats were used because of their higher sensitivity to the development of MASLD than female rats (Lee et al., 2019; Heintz et al., 2020). All rats survived until their scheduled necropsy, and the subcutaneous implantation with hAF or rAF was well tolerated without adverse effects. No abnormalities were noted during observations. At the end of the study, all hAF and rAF were identified at the sites of implantation (Figure 6A). Both hAF and rAF showed similar treatment outcomes. The most significant finding was a reduction of liver steatosis (Figure 6B), which correlated with a statistically significant reduction in hepatic triglycerides after treatment with both hAF and rAF versus the PBS treatment (Figure 6E). After treatment with rAF, the BW decrease reached statistical significance compared to the rats in the PBS-treated group (Figure 6C). Other findings included a trend in reduced body and liver weights (Figures 6C,D). However, the liver-to-BW ratio was not different between the groups in this study, including the baseline group (Figure 6F).

**FIGURE 7:**
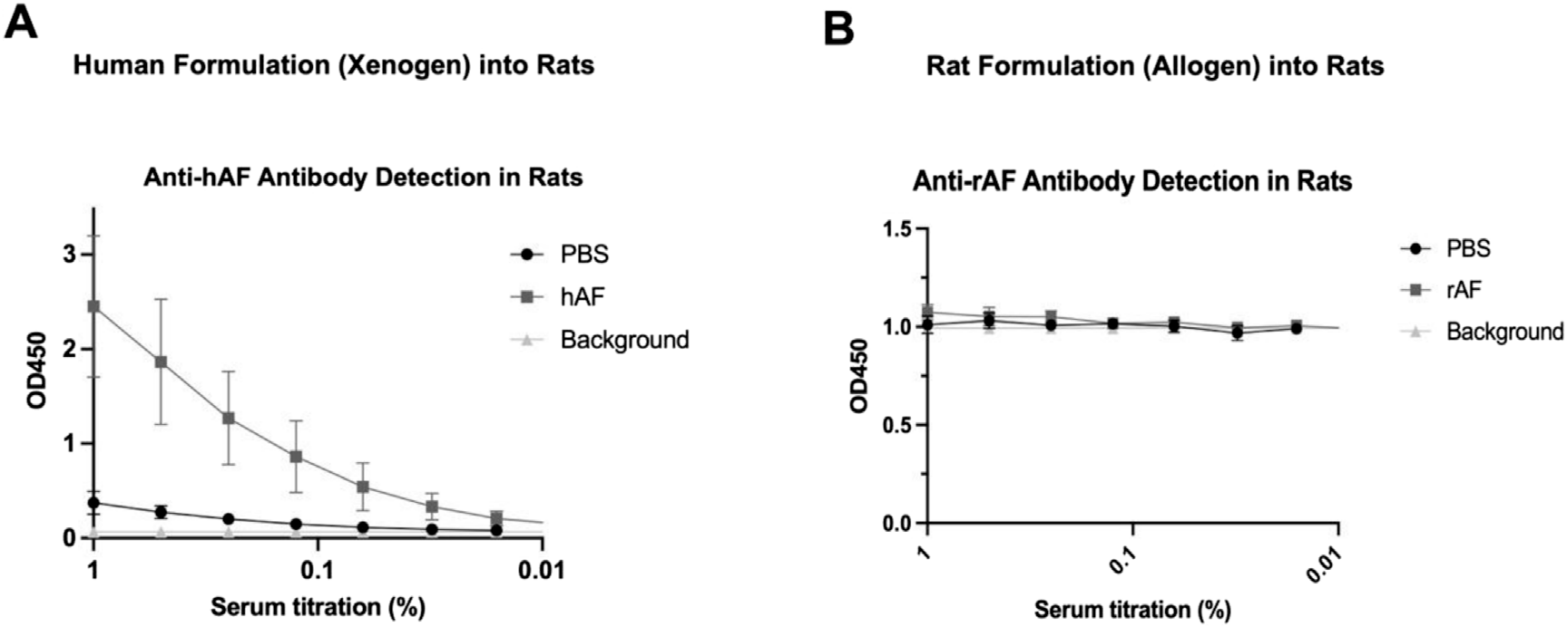
*In vivo* immunogenicity of xenogeneic hAF and allogeneic rAF in rats. Detection of **(A)** anti-hAF or **(B)** anti-rAF antibodies by ELISA coated with hAF or rAF extracts, respectively. Control: serum from rats treated with PBS. Background: no serum. Data are presented as mean ± SD by OD at 450 nm for each serum concentration (n = 6/group).

An immune response against hAF and rAF was evaluated by detection of anti-human and anti-rat AF antibodies in rat serum using ELISA. The results showed that rats developed anti-xenogeneic hAF antibodies at day 28 after the treatment (Figure 7A). In contrast, there were no detectable antibodies in serum from rats treated with allogeneic rAF (Figure 7B). These results indicate that allogeneic rAF, which represents the proposed allogeneic use of hAF in patients, is not immunogenic in rats. Together with immunogenicity testing *in vitro*, this *in vivo* finding confirmed that the developed adipose tissue processing and preservation technology eliminated immunogens, enabling allogeneic use of AF.

### Evaluation of hAF and mAF in a severe MASH with fibrosis model using CGI-58 LivKO mice

**FIGURE 8:**
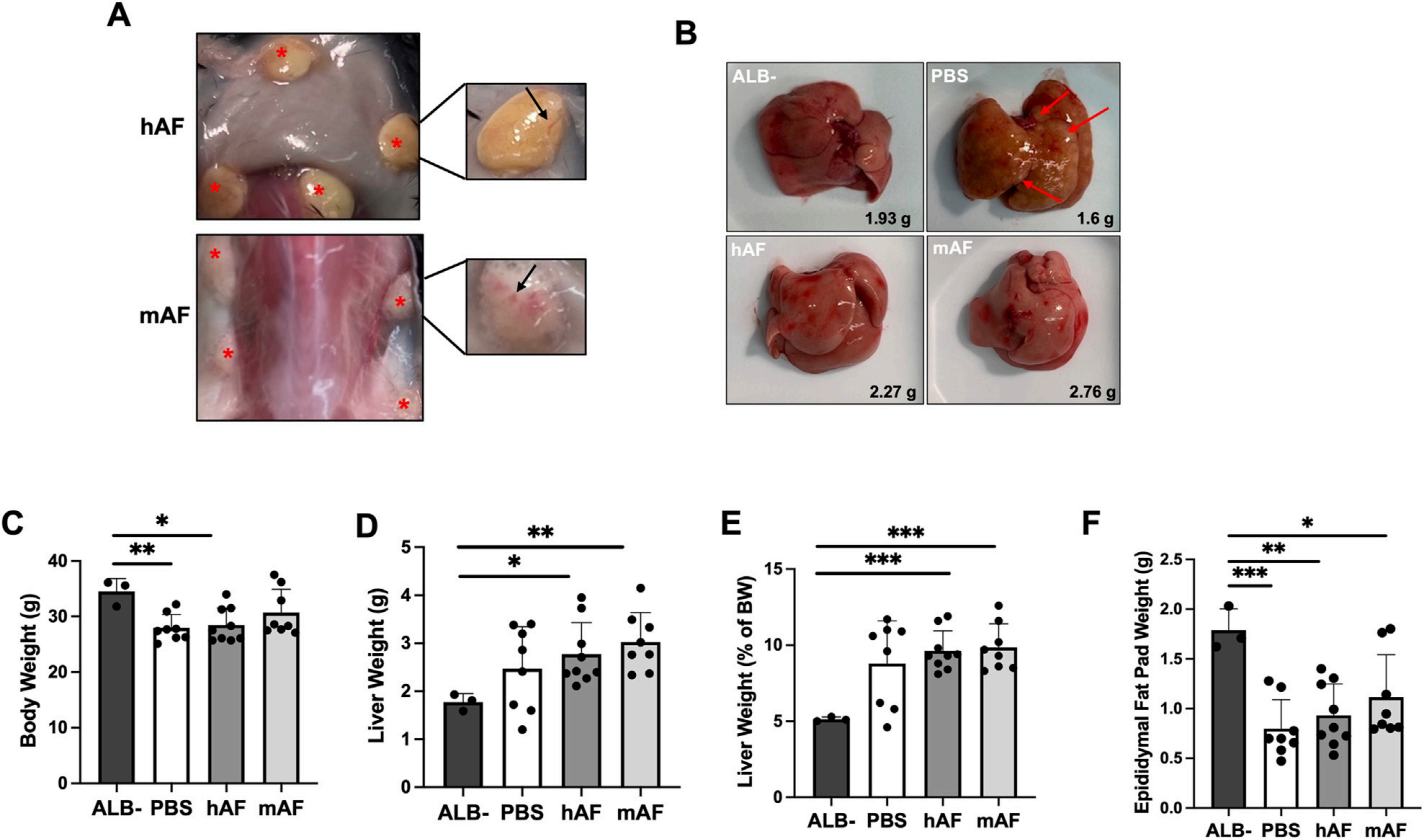
Effects of hAF or mAF treatment on MASH and liver fibrosis in CGI-58 LivKO mice. **(A)** Images of hAF (top) and mAF (bottom) in mice 4 weeks after SC implantation. Arrows point to hAF and mAF. **(B)** Representative images of H&E-stained liver tissue sections in mice. **(C)** Body weights, **(D)** liver weights, **(E)** liver triglycerides (TG), and **(F)** liver/body weight ratio of mice at the end of the study. Data are presented as mean ± SD. n = 8-9 for treatment group, and n = 3 for ALB- group. *P < 0.05 by Student’s t-test.

The objective of this study was to evaluate the therapeutic potential of hAF and mAF to treat MASH and hepatic fibrosis in male CGI-58 knockout (LivKO) mice. Male mice were used because of their higher sensitivity to the development of liver disease than female mice. All mice survived until their scheduled necropsy, and hAF and mAF implantation were well tolerated without adverse effects. No abnormalities were noted during observations. hAF and mAF implants persisted for the duration of the study at the implantation sites (Figure 8A). Figure 8A shows the visual appearance of hAF and mAF at implantation sites after 4 weeks. Both hAF and mAF were imbedded in mouse connective tissue with visible blood vessels formed on the surface of AF formulations (shown in inserts in Figure 8A). The visual appearance of livers is shown in Figure 8B: after PBS treatment, >60% of mice (5 out of 8) had fibrotic nodules in the liver (red arrows), whereas the livers in hAF and mAF-treated mice had no fibrotic nodules and looked healthier, similar to livers in the ALB- control mice (Figure 8B). The BW, liver and epididymal fat pad weights, and liver/BW ratios were significantly different from those in the ALB-control group, but there were no differences between hAF and mAF treated groups versus the PBS group (Figures 8C–F).

**FIGURE 9:**
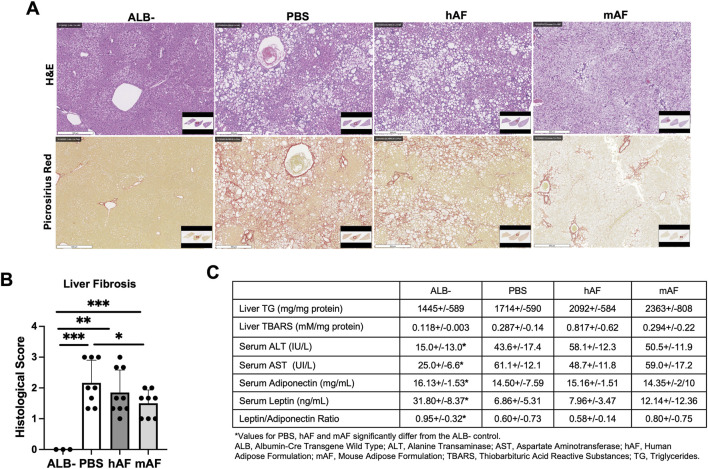
Effects of hAF or mAF treatment on liver fibrosis in CGI-58 LivKO mice. **(A)** Representative images of H&E− or Picrosirius Red-stained liver tissue sections and **(B)** histological score of liver fibrosis. Scale bars: 500 μm. **(C)** Hepatic levels of TG and TBARS and serum parameters. Data are presented as mean ± SD. n = 8-9 for treatment group, and n = 3 for ALB- group. *P < 0.05 by Student’s t-test.

Liver histopathology showed that the mAF group had significantly reduced liver fibrosis (Figures 9A,B). The effect of hAF on fibrosis in mice was significantly weaker when compared to mAF, which can be explained by an immune response against xenogeneic hAF in mice (Figure 7A). Figure 9C summarizes the results of other tests that were performed. Most of the results from those tests showed differences between the ALB-control group versus all treatment groups, although test values after treatment with mAF showed trends toward values in the ALB-control group (Figure 9C).

**FIGURE 10:**
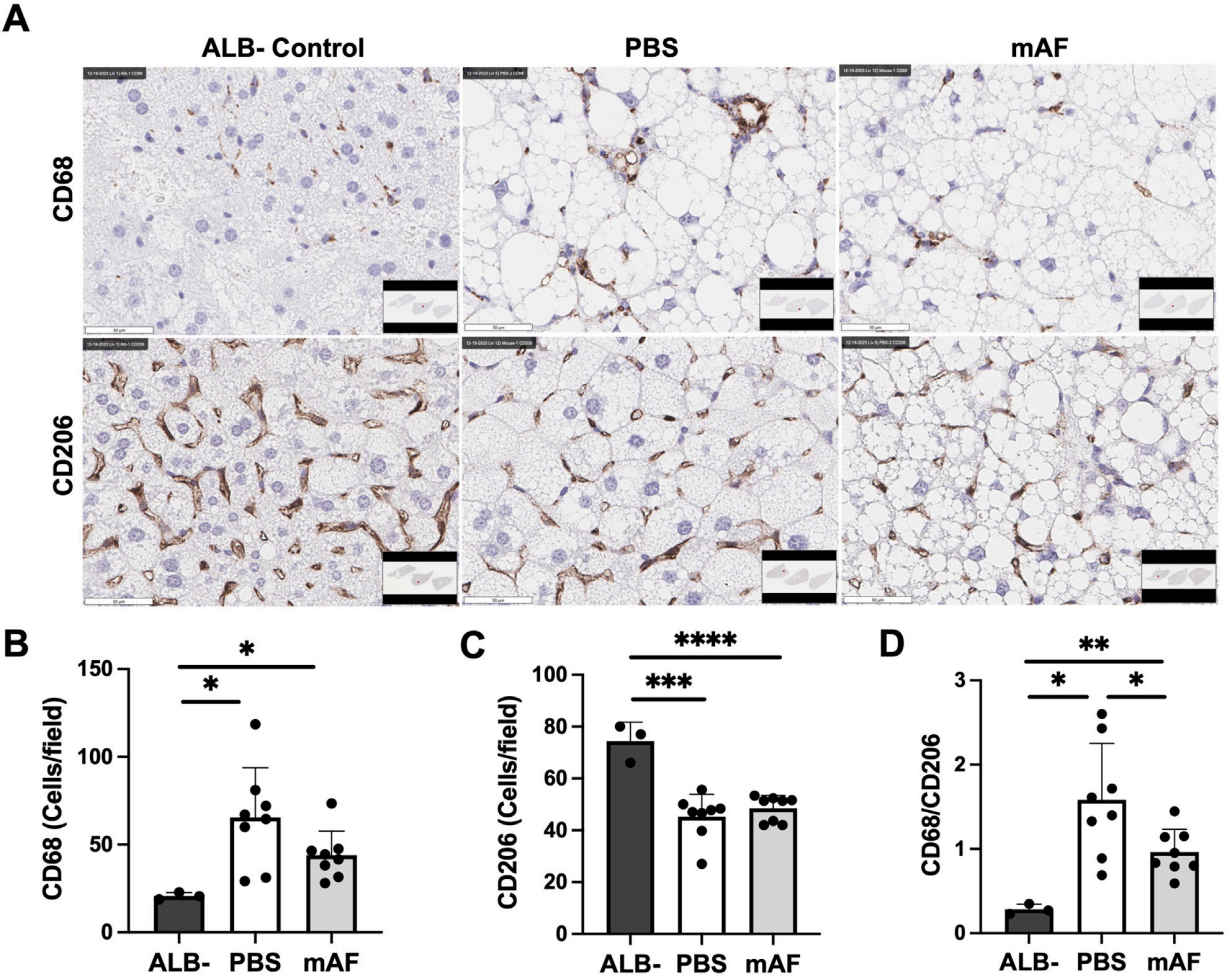
Effects of mAF treatment on hepatic macrophages in CGI-58 LivKO mice. **(A)** Representative IHC images and quantification of the **(B)** M1 macrophage marker CD68, **(C)** M2 macrophage marker CD206, and **(D)** ratio of CD68 to CD206 in the liver of mice. Data are presented as mean ± SD. n = 8 for treatment group, and n = 3 for ALB- group. *P < 0.05, **P < 0.01 by Student’s t-test. Scale bars: 50 μm.

**FIGURE 11:**
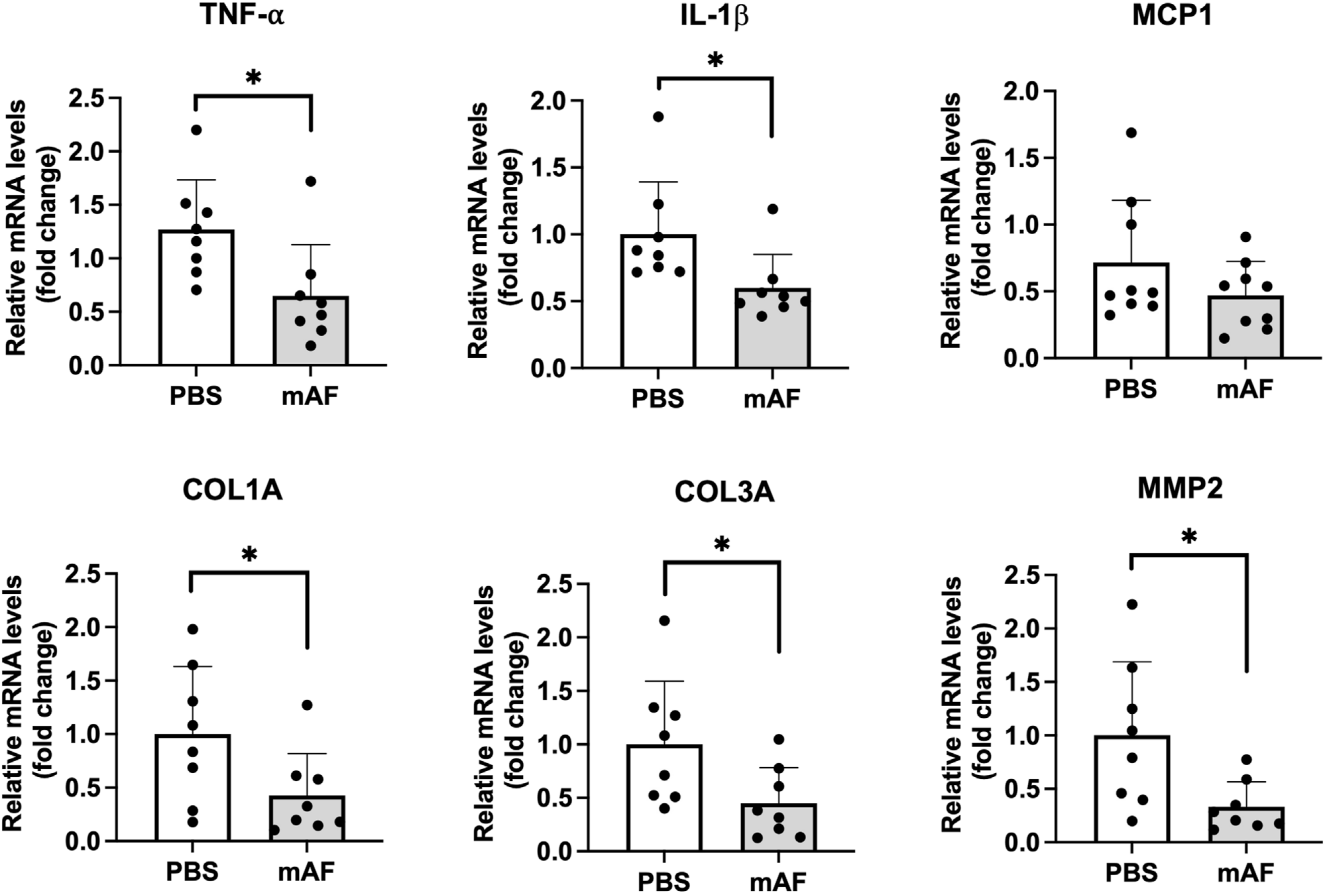
Hepatic mRNA levels of pro-inflammatory and pro-fibrotic genes in the liver of PBS- and mAF-treated CGI-58 LivKO mice. Data are presented as mean ± SEM. n = 8/group. *P < 0.05 by Student’s t-test.

To test a hypothesis that one mechanism of action for mAF involves host macrophages, IHC staining of the M1 (inflammatory) marker CD68 and the M2 marker CD206 in the liver (anti-inflammatory) was performed (Figure 10A). There was a significant reduction in the number of M1 macrophages (CD68 positive cells) in the liver of mAF group when compared to PBS-treated mice (Figure 10B), whereas there was no difference in the number of M2 macrophages (CD206 positive cells) (Figure 10C). However, the decrease in M1 macrophages after mAF treatment resulted in a significant decrease in the M1 to M2 macrophage ratio (Figure 10D). The mRNA levels of pro-inflammatory makers TNF-α and IL-1β were significantly decreased in the mAF group compared to the PBS group (Figure 11). Also, results showed that the mRNA expression of pro-fibrotic genes, including Col1a, Col3a, and MMP2, was downregulated in the mAF-treated mice compared to the PBS-treated mice (Figure 11).

## Discussion

With a low response rate for the only therapy approved by the FDA for MASH in 2024 (Author anonymous, 2024), developing new therapies that can target multiple pathological pathways in MASH remains a high priority. The complex mechanisms of action and data demonstrating positive outcomes in animal and clinical studies make cell- and tissue-based therapies promising candidates for treating MASH (Tsuchiya et al., 2019; Yang P. et al., 2020). In this study, we tested a hypothesis that an allogeneic engineered adipose formulation, comprised of adipose-derived SVF cells combined with adipose tissue particulates, might be beneficial for MASH treatment, similar to the demonstrated benefits of adipose transplantation in animals, but without the risk of immune rejection.

Our culture-expanded adipose SVF cells display characteristics of MSCs because of their spindle-shaped fibroblast morphology, adhesion to plastic surfaces, growth in the MSC-defined medium, and ability to differentiate into mature adipocytes, all of which are recognized properties of MSCs (Dominici et al., 2006; Mushahary et al., 2018). After one passage in culture, the SVF cell population is homogeneous based on the presence of CD90 and CD73 MSC markers and the absence of CD34 and CD45 hematopoietic stem cell (HSC) markers. These cell markerse align with the MSC phenotype described in the literature, with the exception of the CD105 marker (Dominici et al., 2006; Bora and Majumdar, 2017; Mushahary et al., 2018). The SVF cells we used for hAF do not express CD105. However, recent studies indicate the existence of a CD105-negative MSC population in adipose tissue (Levi et al., 2011; Pham et al., 2019). The literature also indicates that CD105 expression changes during culture: it increases from undetectable levels in freshly harvested cells to nearly ubiquitous expression after several days in culture (Baer, 2014); however, CD105 expression remains low when cells are cultured in low or serum-free media (Mark et al., 2013). In this study, adipose-derived SVF cells were cultured in DMEM with low serum, which likely explains the absence of CD105 expression. Functionally, these CD90^pos^CD73^pos^CD105^neg^ SVF cells demonstrated rapid adipogenic differentiation and an increase in VEGF secretion after incubation in a hypoxic chamber, which are known functional characteristics of MSCs (Biniazan et al., 2024).

Allogeneic mesenchymal tissues, such as bone, cartilage, and tendons, have a long and successful history of clinical use with minimal risks of immune-mediated rejection (Beer et al., 2019), attributed to their low immunogenicity (Mazzocca and Lindsay, 2019). Although adipose tissue is a type of mesenchymal tissues, the transplantation of allogeneic adipose tissue in animals results in immune rejection (Ablamunits et al., 2007). Adipose tissue is a highly vascularized tissue, and its immunogenicity is likely linked to the presence of blood cells. Research has shown that freshly isolated SVF cells induce an immune response due to the presence of blood cells, while cultured SVF cells do not (McIntosh et al., 2006). These findings indicate that, similar to other types of mesenchymal tissues, the use of allogeneic adipose tissue should be feasible if immunogenic components are removed. To facilitate the use of allogeneic adipose therapeutic formulations, the development of the adipose tissue processing method has focused on: i) removing immunogenic components, ii) retaining the structure and function of the native adipose tissue, and iii) ensuring tissue injectability for implantation via a minimally invasive injection. The method we developed produces hAPs that are devoid of immunogenic components while retaining the structure of native adipose. In contrast, native adipose tissue expresses multiple inflammatory cytokines (Regulski et al., 2024). As expected, our SVF cells and hAPs secrete significant amounts of VEGF and adipokines (e.g., leptin and adiponectin), respectively, which resembles the native adipose tissue (Ceserani et al., 2016; Yu et al., 2018). VEGF plays a crucial role in the vascularization of the AF after implantation, and adiponectin has anti-inflammatory properties in addition to its metabolic activity (Luo and Liu, 2016; Wang and Scherer, 2016). Using THP-1 cells that are known to respond to LPS with gene transcription and cytokine secretion profiles similar to those of LPS-stimulated PBMC-derived macrophages (Sharif et al., 2007; Schildberger et al., 2013), we demonstrate that hAP, but not SVF cells, exhibited strong anti-inflammatory activity. Histologically, there are no differences between hAP and the native human adipose tissue. Moreover, the hAP can be injected through a 20–21G needle without damaging tissue particles or clogging the needle.

Adipose-derived SVF cells have shown to possess anti-inflammatory and immune-modulating activities (Ceccarelli et al., 2020; Gareev et al., 2024). Our SVF cells have low anti-inflammatory activity. We evaluated the anti-inflammatory activity of cryopreserved SVF cells, hAP, and hAF immediately post-thaw to simulate potential clinical use. The literature presents contradictory data regarding the anti-inflammatory activity of cryopreserved versus cultured adipose SVF cells, with some studies reporting no differences while others indicate a significant decrease in activity of cultured versus cryopreserved cells (Gil-Chinchilla et al., 2024; Wang and Li, 2024). Nevertheless, the most significant finding of our study was that the devitalized cryopreserved human and rodent APs exhibit strong anti-inflammatory activity. A comparison of human APs to decellularized adipose tissue, the most common component used in tissue engineering as reported in the literature, showed that decellularized adipose tissue has significantly decreased anti-inflammatory activity (unpublished data). This result can be explained by the “washing out” of anti-inflammatory factors present in the tissue during the decellularization process.

Although the current expert opinion suggests that the therapeutic effects of adipose tissue are primarily due to SVF stem cells within it, direct confirmation is lacking. This view relies on both clinical and nonclinical studies demonstrating the therapeutic benefits of culture-expanded adipose-derived SVF cells (Issa et al., 2022; Yan et al., 2022). However, results from this study indicate that the anti-inflammatory effect of hAF is attributed to the devitalized hAP rather than the SVF cells. Our finding is consistent with previous studies demonstrating the anti-inflammatory effects of cell-free adipose tissue extracts in Raw 264.7 macrophages and a rat model of osteoarthritis (Jia et al., 2022). Furthermore, two additional studies have observed the anti-inflammatory activity of conditioned media derived from micro-fragmented adipose tissue (MFAT) (Ceserani et al., 2016; Nava et al., 2019). Kokai and colleagues evaluated MFAT, emulsified adipose tissue (nanofat), and enzymatically isolated SVF cells in *in vitro* co-cultures with primary human articular chondrocytes. Their results showed that chondrocytes cultured with SVF cells exhibited increased inflammatory gene expression, whereas those co-cultured with either nanofat or MFAT tissue displayed minimal pro-inflammatory effects. These findings led to the conclusion that mechanically processed adipose preparations may be more effective than isolated SVF cells (Kokai et al., 2022).

The therapeutic effects of hAF were assessed in two rodent models of MASLD. Given that hAF, a highly immunogenic xenogen (Badylak and Gilbert, 2008), is expected to elicit an immune response in animals, the evaluation of allogeneic rAF and mAF surrogates was also incorporated into the animal studies. Although rAF and mAF are not clinical formulations suited for patient treatment, allogeneic rAF and mAF serve as models for the potential clinical application of allogeneic hAF. In the obese Zucker model, we only observed hepatic steatosis without apparent inflammation. And fibrosis, even after 18 weeks of high-fat and high-cholesterol feeding (data not shown), which was different from what was reported by Matsumoto and colleagues—Obese Zucker rats developed the full spectrum of MASH pathologies after 4–12 weeks on a similar high-fat and high-cholesterol diet (Matsunami et al., 2010). The discrepancy may stem from variations of obese Zucker rats from different suppliers across different countries, which was reported to significantly influence study outcomes (Colman, 2017). It has been previously reported that the implantation of poly (lactide-co-glycolide) scaffolds into the epididymal fat of mice on a high-fat diet protected against hyperinsulinemia and ectopic fat accumulation in the liver and skeletal muscles; and the positive effects correlated with increased CPT1A enzyme expression in multinucleated giant cells surrounding the implant, which catabolizes fatty acids (Hendley et al., 2020). Potentially, similar mechanisms may mediate the effects of hAF and rAF in reducing lipid accumulation in the liver.

The second model involved severe MASH induced by a Western diet in liver-specific CGI-58 knockout (LivKO) mice. Mutations in human CGI-58, also known as α/β hydrolase domain-containing 5 (Abhd5), lead to Chanarin-Dorfman syndrome (CDS), a rare autosomal recessive neutral lipid storage disease characterized by ichthyosis and the accumulation of TG-rich cytosolic LDs in all cell types (Lefevre et al., 2001; Lass et al., 2006). LivKO mice fed a Western diet quickly develop the full spectrum of MASH pathologies (Yang X. et al., 2020). Interestingly, treating LivKO mice with mAF led to a significant reduction in hepatic expression of genes related to inflammation and fibrosis, though having no impact on hepatic steatosis. The limited effectiveness of hAF may be attributable to an immune response against xenogeneic hAF, which can increase inflammation and mask the beneficial effects of hAF in mice. The level of TBARS, an oxidative stress marker, in the livers after hAF treatment was significantly higher than that in the other groups, supporting this speculation. Moreover, structural differences between mouse and human growth factors and cytokines may result in reduced biological activity of human molecules in mice. Furthermore, SVF cells were observed to be the primary source of VEGF-A, an angiogenesis factor that supports the proliferation of sinusoidal endothelial cells and hepatocytes during liver regeneration (Bockhorn et al., 2007). A previous report (Choi et al., 2019) demonstrated that SVF culture medium inhibited hepatic stellate cell activation *in vitro*, resulting in lower mRNA levels of Col1A1 and MMP-2 following TGF–β treatment, while the levels of hepatocyte growth factor, VEGF-A, IL-10, and microRNA-451 were higher in SVF compared to human adipose-derived mesenchymal stem cells (ASCs). Furthermore, the injection of SVF via the portal vein significantly reduced thioacetamide-induced liver fibrosis, with lower mRNA levels of IL-1β, IL-6, and TNF-α compared to the ASC and PBS-injected groups. These findings indicate that human adipose-derived SVF has the ability to upregulate hepatocyte growth and exhibit notable anti-inflammatory effects for treating fibrotic liver diseases through IL-10 secretion.

Treatment with hAF and mAF did not affect serum ALT and AST levels (Figure 9C). While elevated ALT and AST often indicate liver cell injury, their levels do not always directly correlate with the extent of fibrosis (Fracanzani et al., 2008). Treatment with hAF and mAF is likely to effectively reduce liver fibrosis without significantly impacting the level of liver cell damage. In the pathophysiological context of MASLD, it has been consistently observed that elevated serum leptin levels coincide with reduced adiponectin concentrations, indicating that an increased leptin-to-adiponectin ratio (LAR) could potentially function as a biomarker for MASLD severity. Specifically, heightened leptin and diminished adiponectin levels correlate with a heightened risk for the development of MASLD (Marques et al., 2021). We observed that adiponectin, leptin, and LAR across all treatment groups were significantly reduced as compared to the ALB-control group. This reduction is more closely associated with decreased adipose mass observed in the LivKO mice, and the relatively shorter half-life of leptin compared to that of adiponectin in terms of LAR (Halberg et al., 2009; Burnett et al., 2017).

Reduced numbers of infiltrated CD68-positive macrophages were detected in the mAF-treated group compared with the PBS-treated group. Macrophages are crucial in the progression of MASLD (Lefere and Tacke, 2019). Liver macrophages are heterogeneous, contributing to liver homeostasis and the progression or regression of liver disease (Tacke and Zimmermann, 2014). The origin of the macrophages is closely associated with their replenishment and activation during liver fibrosis. Hepatic macrophages originate from either circulating monocytes recruited to the injured liver via chemokine signals or local Kupfer cells (KCs). Inflammatory stimuli and metabolic signals may activate KCs (CD68^+^ macrophages) to produce proinflammatory cytokines and chemokines. This can lead to the recruitment of other inflammatory cells and the trans-differentiation of hepatic stellate cells into myofibroblasts, the primary collagen-producing cell type contributing to hepatic fibrosis (Tacke and Zimmermann, 2014). The pro-fibrotic role of macrophages in liver fibrosis is well-established (Ramachandran et al., 2012), and research has focused on targeting these cells as a therapeutic strategy for liver fibrosis (Jiang et al., 2024). In the early stages of liver fibrosis, the activation of hepatic stellate cells by macrophages through inflammatory cytokines represents a key pathway that exacerbates liver fibrosis by promoting the production of extracellular matrix proteins like collagen, ultimately resulting in scar tissue formation (Barnes et al., 2015). Additionally, macrophages facilitate fibrosis by releasing mediators such as profibrogenic cytokines transforming growth factor-β and PDGF, which trigger hepatic stellate cells (Dooley and ten Dijke, 2012; Kocabayoglu et al., 2015). Monocyte-derived macrophages that are positive for the stimulator of interferon genes (STING) can also contribute to hepatic inflammation and fibrosis progression. STING is primarily expressed in monocyte-derived macrophages, KCs, and CD163+ macrophages, and the number of STING + hepatic macrophages significantly increases in MASH, correlating with the worsening of fibrosis (Wang et al., 2020). On the other hand, recently, a macrophage subpopulation that is positive for a liver lipid-associated triggering receptor expressed on myeloid cells-2 (TREM2) has been identified as critical for resolving MASH/fibrosis (Ganguly et al., 2024). Efficient collagen degradation is proposed as an underlying mechanism for resolving hepatic fibrosis by TREM2+ macrophages (Ganguly et al., 2024).

The decrease in liver fibrosis correlated with a notable reduction in hepatic inflammatory M1 macrophages, a lower ratio of M1 to M2 macrophages, and decreased inflammatory gene expression in the liver. There was no increase in M2 macrophages following mAF treatment. The biodistribution evaluation of hAF and rAF in naïve mice and rats showed that no detectable human molecules were present in systemic blood circulation (data not included in this study), which indicates that the mechanisms of action do not involve the release of anti-inflammatory factors from the implanted AFs. The results of this study, in conjunction with existing literature, suggest that following implantation, mAF may promote the maturation of blood monocytes into anti-inflammatory macrophages, allowing them to migrate into the liver and resolve fibrosis. Testing of this hypothesis is ongoing, and results will be reported separately.

In conclusion, the primary finding of this study is that in a model of severe MASH, treating animals with the engineered AFs prevents the progression of hepatic inflammation and fibrosis. Whether engineered AFs can reverse an advanced stage of the disease remains to be determined in future studies. While the results show the safety and potential efficacy of the engineered AFs, several key questions remain unanswered before moving to the clinical phase of development. These questions pertain to the optimization of the AF composition, the therapeutic dose and treatment regimen, and the mechanisms behind the anti-inflammatory and anti-fibrotic effects of AFs.

## Data Availability

The raw data supporting the conclusions of this article will be made available by the authors, without undue reservation.
