# Correction: An engineered adipose formulation decreases hepatic inflammation and fibrosis in a rodent model of metabolic dysfunction-associated steatotic liver disease

**DOI:** 10.3389/fbioe.2025.1642526

**Published:** 2025-06-26

**Authors:** Youngshim Choi, Yinyan Ma, Samson Tom, Alla Danilkovitch, Liqing Yu

**Affiliations:** ^1^ Division of Endocrinology, Diabetes and Nutrition, Department of Medicine, University of Maryland School of Medicine, Baltimore, MD, United States; ^2^ Britecyte, Inc., Fredrick, MD, United States

**Keywords:** adipose tissue, adipose stem cells, tissue engineering, MASLD, fibrosis, inflammation

In the published article, there was an error in Figure 7B as published. The graph in Figure 7B was identical to Figure 7A, and has been replaced with the correct version. The corrected Figure 7B and its caption “FIGURE 7: In vivo immunogenicity of xenogeneic hAF and allogeneic rAF in rats. Detection of (A) anti-hAF or (B) anti-rAF antibodies by ELISA coated with hAF or rAF extracts, respectively. Control: serum from rats treated with PBS. Background: no serum. Data are presented as mean ± SD by OD at 450 nm for each serum concentration (n = 6/group).” appear below.

**FIGURE 7:**
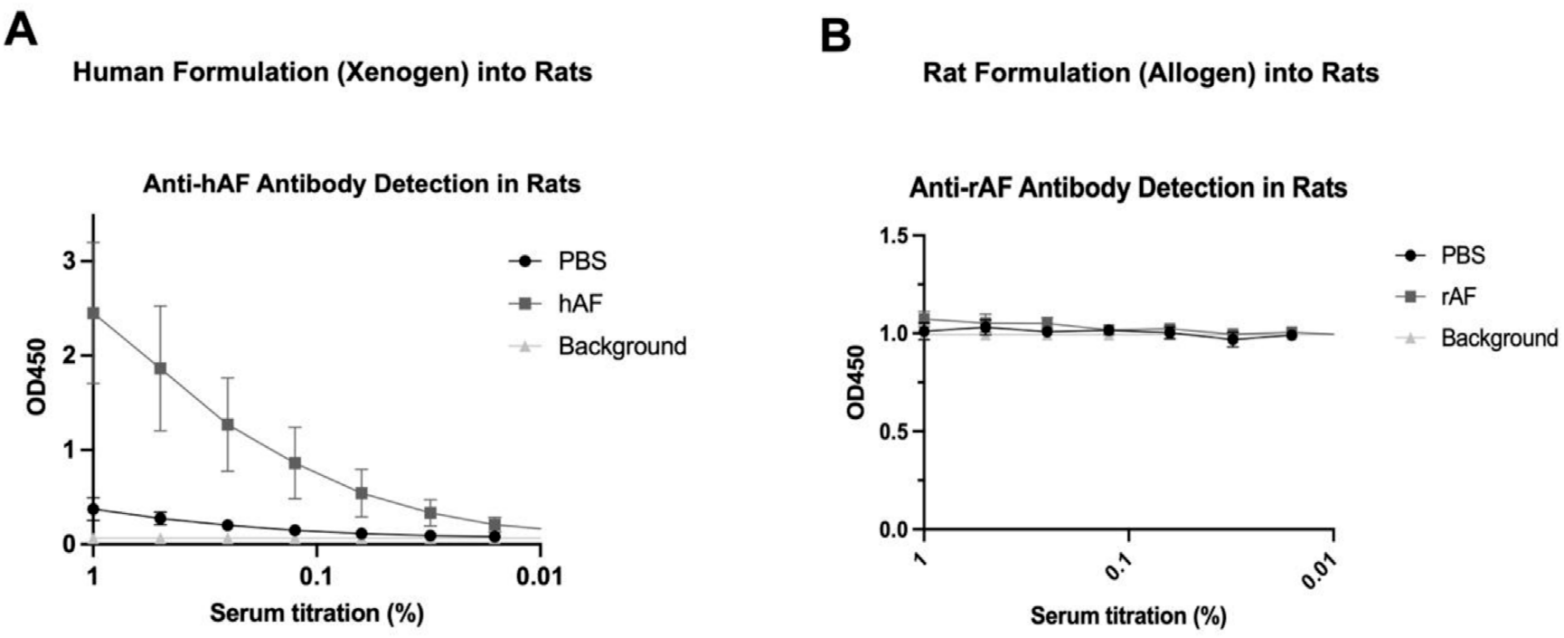
*In vivo* immunogenicity of xenogeneic hAF and allogeneic rAF in rats. Detection of **(A)** anti-hAF or **(B)** anti-rAF antibodies by ELISA coated with hAF or rAF extracts, respectively. Control: serum from rats treated with PBS. Background: no serum. Data are presented as mean ± SD by OD at 450 nm for each serum concentration (n = 6/group).

The original version of this article has been updated.

